# Ultrasound-Guided Lauromacrogol Injection for the Treatment of Active Bleeding After Renal Biopsy

**DOI:** 10.3389/fphar.2021.723634

**Published:** 2021-12-23

**Authors:** Weizong Liu, Chunchun Jin, Qingshu Lian, Lifeng Xu, Zhanye Lin, Jianghao Lu, Xuehao Gong

**Affiliations:** Department of Ultrasound, The First Affiliated Hospital of Shenzhen University, Shenzhen Second People’s Hospital, Shenzhen, China

**Keywords:** ultrasound, lauromacrogol injection, renal, biopsy complication, bleeding

## Abstract

**Background:** This study aimed to describe the technique and outcomes of hemostasis for ultrasound-guided lauromacrogol injection for active bleeding after renal biopsy.

**Methods:** Data from patients with active bleeding after renal biopsy between January 2018 and December 2020 were retrospectively collected. Patients who still had active bleeding after 30 min of compression were then injected with lauromacrogol under ultrasound guidance. The patient’s symptoms before and after operation were collected to assess whether they had severe complications. Changes in hemoglobin and serum creatinine values were collected.

**Results:** Data from a total of 15 patients with active bleeding after renal biopsy were collected, including data of 6 men and 9 women. After the operation, there were 11 cases of mild back pain; 1 case of chills, cold sweats, and back pain; 1 case of cold sweats and blood pressure reduction, and 2 cases with no obvious symptoms. No severe complications occurred in this study, and active bleeding was stopped in all patients. After the operation, compared with before the operation, there was no statistically significant difference in the hemoglobin value and serum creatinine value (*p* = 0.10 > 0.05, *p* = 0.78 > 0.05).

**Conclusion:** Ultrasound-guided lauromacrogol injection is a relatively simple, safe and feasible method, which could be helpful in treating active bleeding in the immediate post-procedure period after renal biopsy.

## Introduction

Ultrasound-guided renal biopsy is currently one of the routine examination procedures in the nephrology department for pathological analysis and to determine precise treatment; however, it is an invasive examination. Bleeding is a complication of renal biopsy (Korbet et al., 2014; Preuss et al., 2017; Shen and Ro, 2019), with an incidence of 5–7%. Most cases can be relieved by conservative treatment, but 0.5% of patients still have poor results after conservative treatment, with severe hemorrhagic shock requiring surgery or interventional treatment (Bakdash et al., 2019; Prasad et al., 2015; Tøndel et al., 2012; Whittier and Korbet, 2004). Interventional therapy has the advantages of timely positioning of abnormal blood vessels and blocking under angiography (Somani et al., 2006). However, the patient needs to be taken to the angiography operating room for embolization, which may cause delays in treatment. In addition, the iodine contrast agent used in angiography has shortcomings such as allergic reactions and acute kidney injury. After embolization there might be complications such as postembolization syndrome, infection, and renal insufficiency (Dinkel et al., 2002; Onuigbo et al., 2020; Sayani et al., 2012). Postembolization syndrome, which is the most common complication, can affect up to 90% of patients (Ginat et al., 2009). Furthermore, embolization treatment is time-consuming and expensive. Therefore, it is necessary to develop a treatment with fewer adverse reactions that is simple, safe, and feasible.

Lauromacrogol injection is a sclerosing agent that has certain local anesthesia, adhesion, and hemostatic effects. It has been successfully used in sclerotherapy for varicose veins, cysts, and vascular malformations. Its operation is simple, fast and less time-consuming. The application of lauromacrogol injection has a good curative effect and few side reactions (Xue and Geng, 2015; Liu L. et al., 2016; Chen et al., 2016; Dong et al., 2019; Rosa, 2019). However, to the best of our knowledge, lauromacrogol injection has not been reported in the treatment of active bleeding after renal biopsy. Therefore, this study aimed to describe the technique and outcomes of hemostasis for ultrasound-guided lauromacrogol injection for active bleeding after renal biopsy.

## Materials and Methods

### Study Patients

Data from patients who underwent ultrasound-guided renal biopsy between January 2018 and December 2020 were retrospectively collected. All patients had native kidneys and not kidneys transplants. Patients who underwent color Doppler ultrasound that showed no active bleeding after 30 min of compression and had incomplete clinical data were excluded. Patients who underwent color Doppler ultrasound that showed active bleeding after 30 min of compression were included in this study. This retrospective study was approved by the Ethics Committee of our hospital.

### Preoperative Preparation

Firstly, routine blood tests were performed on the patients. Blood pressure was controlled for hypertensive patients to below 140/90 mmHg. We retrospectively collected the bleeding time, prothrombin time, liver, and kidney function, etc., and confirmed that the patient’s coagulation function was within the normal range and there was no contraindication to the operation. Patients were then instructed to empty their urine and bowels, andmenstrual periods were avoided in female patients. Written informed consent was routinely obtained from each patient before the biopsy, so all patients were fully informed of the advantages and disadvantages of the following operation.

### Equipment and Operational Tools

Ultrasound examinations and renal biopsies were conducted using Mylab90 and Twice (Esaote, Genoa, and Italy). A convex array probe (CA541) with 1.0–8.0 MHz was used for conventional ultrasound examinations. The puncture frame matched with the convex array probe was used to guide the puncture. Lidocaine injection was administered before the biopsy. Kidney tissue was obtained by core biopsy using a Bard® Max-Core® Disposable Core Biopsy Instrument (#MC1616; Bard Biopsy Systems, AZ, United States ), and the ejection distance was 22 mm. A 22-gauge PAN needle, the cell biopsy needle (Gallini Medical Devices, size 22G 15 cm, Mantova, and Italy), was used to inject the lauromacrogol (lauromacrogol injection, 10 ml: 100 mg; Tianyu Pharmaceutical Co., Ltd., Shanxi, China), which was slowly injected at active bleeding points at a rate of about 0.2 ml/s after renal biopsy.

### Ultrasound Examination

All patients were treated by a doctor with 15 years of puncture experience. Before renal biopsy, conventional ultrasound examinations were performed to observe and measure the location, shape, size, and renal parenchyma thickness, to understand the patient’s kidney activity, and to determine whether there were stones in the renal parenchyma or space-occupying lesions. Color Doppler ultrasound was performed to observe the blood supply to the kidneys.

### Ultrasound-Guided Renal Biopsy and Lauromacrogol Injection

Patients were placed in the prone position with their arms facing up and head tilted to one side. A 5–10 cm thick cotton pillow was placed on the abdomen. Simultaneously, an abdominal band was placed under the abdomen to prepare for postoperative compression and bandaging. Routine ultrasound was used to scan the kidneys to determine the location and route of the biopsy. The thicker renal parenchyma at the lower extreme lateral edge of the right kidney was usually chosen as the puncture target. If the lower pole of the right kidney was blocked by the ribs, intestines, etc., the lower pole of the left kidney was chosen. After determining the biopsy target, patients were asked to inhale deeply and hold their breath to ensure that the kidney could be fixed for at least 3 s and positioned on the body surface. The depth of the skin from the renal capsule was measured in the breath-hold state. Local anesthesia was performed with 5 ml of 2% lidocaine, starting from the skin layer and stopping at the renal capsule. Under ultrasound guidance, a 16-gauge needle was inserted through the marked puncture point. When the tip of the biopsy needle reached outside the renal capsule, patients were asked to inhale deeply and maintain a breath-holding state. The biopsy needle was quickly pierced into the renal capsule to reach the lower pole of the kidney. The biopsy device was used to take the specimen 1–2 times, which must be larger than 10 mm, and then the biopsy device was removed. The specimens were fixed with 10% formaldehyde and sent to the pathology department for pathological examination.

After the needle biopsy, color Doppler ultrasound was used to observe active bleeding at the puncture site. After 30 min of local compression, color Doppler ultrasound was performed again. If there was still active bleeding at the puncture site, the 22-gauge PAN needle was punctured to the bleeding point of the renal capsule under the guidance of color Doppler ultrasound, and lauromacrogol injection was slowly injected at active bleeding points at a rate of about 0.2 ml/s, until the color Doppler ultrasound showed that the blood flow signal disappeared. After the operation, the puncture point was locally compressed, and the abdominal band was bandaged.

### Follow-Up Procedure

After renal biopsy, all patients were in the supine position. Vital signs were continuously monitored for 24 h and whether the patients had adverse reactions were collected. The hemoglobin and serum creatinine values within 2 h after the operation were collected.

### Statistical Analysis

Statistical analyses were performed using SPSS version 20.0 (IBM Corp., Armonk, NY, United States). The paired t-test was performed to analyze differences in mean hemoglobin and creatinine values before and after operation. A *p*-value of <0.05 was considered statistically significant.

## Results

**FIGURE 1:**
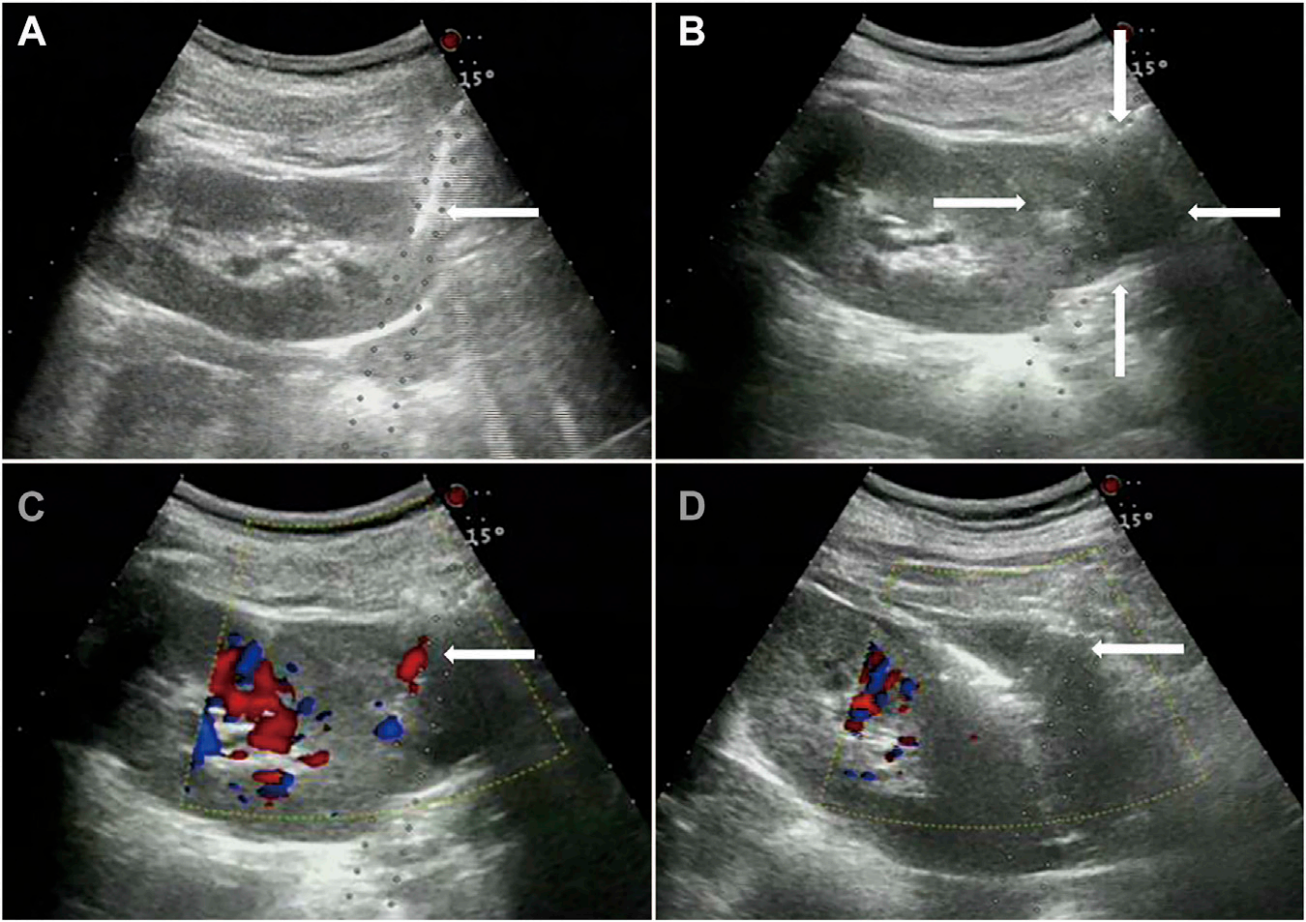
Glomerular disease in a 63-year-old woman. **(A)**, Core needle biopsy of renal parenchyma. **(B)**, Hematomas can be seen around the kidney. **(C)**, 30 min after the biopsy, active bleeding within the biopsy track indicated by color Doppler. **(D)**, 22-gauge PAN needle was punctured to the bleeding point of renal capsule under the guidance of color Doppler ultrasound, and after 4 ml lauromacrogol injection, the color Doppler ultrasound showed that the blood flow signal disappeared.

Data from 15 patients (6 male and 9 female, average age 39 ± 18 years). The pathological diagnosis was glomerular disease in 11 cases, ANCA-associated vasculitis in 2 cases, and lupus nephritis in 2 cases (Table 1). After injection, there were 11 cases of mild back pain, 1 case of chills, cold sweats, and back pain, 1 case of cold sweats and blood pressure reduction, and 2 cases with no obvious symptoms. No serious adverse reactions were observed. The mean dose ±SD of lauromacrogol injection was 3.8 ± 1.4 ml (range, 2–7 ml). All patients with active bleeding successfully stopped bleeding after lauromacrogol injection (Figure 1).

**TABLE 1:** Clinicopathological data of the patients, *n* = 15.

Sex	Age, year	Dosage, ml	Pathological type	Symptom	Scr (μmol/L)	Scr (μmol/L)	Hb (g/L)	Hb (g/L)
					Before operation	After operation	Before operation	After operation
F	35	3	Glomerular disease	Back pain	166.1	165.0	145.0	118.0
M	18	3	Glomerular disease	Back pain	102.0	87.2	142.0	132.0
F	27	5	Glomerular disease	Back pain	48.8	41.9	104.0	103.0
M	27	5	Glomerular disease	Back pain	143.9	134.5	156.0	147.0
M	63	5	ANCA-associated vasculitis	No	359.8	335.2	73.0	72.0
F	26	3	Lupus nephritis	Back pain	113.4	188.7	97.0	90.0
F	31	3	Glomerular disease	Back pain	88.8	99.9	93.0	100.0
M	59	3	Glomerular disease	No	62.0	61.2	126.0	144.0
F	23	5	Lupus nephritis	Chills/cold sweats/Back pain	324.0	433.0	68.0	61.0
F	43	5	Glomerular disease	Back pain	49.3	49.3	115.0	114.0
F	27	2	Glomerular disease	Cold sweats/Blood pressure drop	54.0	47.0	125.0	114.0
M	23	2.5	Glomerular disease	Back pain	168.2	155.0	130.0	119.0
M	76	7	ANCA-associated vasculitis	Back pain	557.0	576.1	76.0	72.0
F	66	4	Glomerular disease	Back pain	83.7	93.0	80.0	78.0
F	39	2	Glomerular disease	Back pain	46.0	53.5	81.0	80.0

(Scr: Serum creatinine, Hb: hemoglobin).

Before the operation, the patient’s average hemoglobin level was 107.4 g/L (median: 104.0 g/L, range: 68.0–156.0 g/L, standard deviation (SD): 28.9 g/L, 95% confidence interval (CI): 91.4–123.4 g/L). The average hemoglobin level within 2 h after the operation was 102.9 g/L (median: 103.0 g/L, range: 61.0–147.0 g/L, and SD: 27.0 g/L, 95% CI: 88.0–117.9 g/L). The change in the average hemoglobin level before and after the operation was not statistically different (*p* = 0.10 > 0.05).

Before the operation, the average serum creatinine level was 157.8 μmol/L (median: 102.0 μmol/L, range: 46.0–557.0 μmol/L, and SD: 146.3 μmol/L, 95% CI: 76.8–238.8 μmol/L). The average serum creatinine level within 2 h after operation was 168.0 μmol/L (median: 99.9 μmol/L, range: 42.0–576.0 μmol/L, and SD: 158.7 μmol/L, 95% CI: 80.2–255.9 μmol/L). There was no statistically significant difference in the change in the average blood creatinine levels before and after operation (*p* = 0.78 > 0.05).

## Discussion

Ultrasound-guided biopsy is currently a widely used renal biopsy technique in clinical practice for its characteristic of being a simple operation with high accuracy; however, it is still an invasive examination with bleeding and other related complications, and can even be life-threatening (Korbet et al., 2014; Preuss et al., 2017). Renal artery embolization can be used to treat bleeding after renal biopsy, but it has complications such as post-embolization syndrome, infection, and renal insufficiency (Dinkel et al., 2002). Therefore, this study proposed to explore the safety and feasibility of ultrasound-guided lauromacrogol injection in treating active bleeding after renal biopsy.

After renal biopsy, local compression was performed for 30 min. Color Doppler ultrasound once again showed the presence of active bleeding, and lauromacrogol was injected immediately into the bleeding point of the renal capsule. After the operation, there were 11 cases of mild back pain, 1 case of chills, cold sweats, and back pain, 1 case of cold sweats and blood pressure reduction, and 2 cases with no obvious symptoms. Mild back pain may be caused by damage to the patient’s tissues, organs, and skin (Haochen et al., 2019). Sweating, nausea, and lowered blood pressure may be caused by the patient’s nervousness and vagus nerve reflex, caused by the stimulation of the nerves of the renal capsule by the biopsy (Tanaka and Okusa, 2020). When the patients rested supine for 30 min, their symptoms were relieved. Due to the short interval between lauromacrogol injection and biopsy, patient’s mild adverse reactions could have been caused by the renal biopsy, lauromacrogol injection, or a combination of the biopsy and lauromacrogol injection. However, it is obvious that the patients showed only mild side effects, and that no obvious serious side effects occurred after lauromacrogol injection. Previous studies have shown that lauromacrogol injection has been used to treat varicose veins, cysts, and vascular malformations, with few side reactions and a good curative effect (Xue and Geng, 2015; Liu W. et al., 2016; Chen et al., 2016; Dong et al., 2019). Therefore, ultrasound-guided lauromacrogol injection in patients with active bleeding after renal biopsy is a simple and fast method, without severe adverse reactions.

Active bleeding after renal biopsy generally stops within a few seconds after the ultrasound-guided lauromacrogol injection. The interval is very short. For example, after reviewing the operation records, it was found that seven of the patients were recorded as follows: 2, 5, 7, 9, 6, 5, and 17 s. In this study, 15 patients with active bleeding after renal biopsy were successfully treated with lauromacrogol injection. Ultrasound-guided lauromacrogol injection can quickly identify the bleeding location, allowing the drug to act directly on the lesion in a short time, destroying vascular endothelial cells, promoting thrombosis, stimulating the formation of a fibrous tissue-protective layer around the ruptured blood vessel, enhancing vascular resistance, slowing blood flow speed, and promoting blood vessel protection, to achieve hemostasis (Liu L. et al., 2016). Before and after operation, there was no significant change in hemoglobin level (*p* = 0.10 > 0.05), indicating that ultrasound-guided lauromacrogol injection is a safe and feasible method for treating active bleeding. In addition, there was no significant change in blood creatinine levels before and after operation (*p* = 0.78 > 0.05), indicating that lauromacrogol injection did not impair renal function.

It is reported that ultrasound-guided injections of thrombin can also be useful in the treatment of active renal bleeding (Mark and William, 2021). Thrombin is a serine protease responsible for converting fibrinogen to fibrin and has demonstrated utility in several applications involving vascular complication. When thrombin is used for active bleeding after renal biopsy, a large dose of thrombin is injected into the perinephric space immediately adjacent to the bleeding site to stop bleeding, instead of a direct injection of thrombin into the blood vessels. If thrombin is injected into the blood vessel by mistake, it can cause thrombosis, local necrosis and even be life-threatening. Lauromacrogol has also been widely used in varicose veins and other fields, and has a good clinical therapeutic effect (Xue and Geng, 2015; Liu W. et al., 2016; Chen et al., 2016; Dong et al., 2019; Rosa, 2019). In this study, the dosage range of lauromacrogol used was 2–7 ml. Lauromacrogol can be injected directly into the local bleeding point, without high-dose injection, and the hemostatic effect can act quickly and efficiently.

The study has several limitations given the relatively small number of patients. First, inherent bias and variations are inevitable because this was a retrospective study. Second, local compression was performed for only 30 mins for patients with active bleeding. Thus, it’s uncertain whether the bleeding would have stopped if we had pressed longer. This is because a continuous active bleeding would receive positive intervention to prevent massive bleeding in clinical work. By reviewing the examination results of these patients 2 h later, we found that there was no delayed bleeding in 15 cases. There was, however, a lack of long-term follow-up to assess rebleeding since assessment of active bleeding at 2 h after injection might have missed delayed bleeding in some cases. The strength of the study, therefore, is that a novel method was performed to control post-renal biopsy bleeding.

## Conclusion

In conclusion, this preliminary study demonstrates that ultrasound-guided lauromacrogol injection is simple, safe and feasible for the treatment of active bleeding on renal biopsy. The successful attempt of lauromacrogol injection for active bleeding after renal biopsy has expanded its application range and has the advantages of relatively low risk, easy operation, and low cost.

## Data Availability

The original contributions presented in the study are included in the article/Supplementary Material, further inquiries can be directed to the corresponding author.
